# Time-Variant Positive Air Pressure in Drainage Stacks as a Pathogen Transmission Pathway of COVID-19

**DOI:** 10.3390/ijerph18116068

**Published:** 2021-06-04

**Authors:** Ling-Tim Wong, Kwok-Wai Mui, Cheng-Li Cheng, Polly Hang-Mei Leung

**Affiliations:** 1 Department of Building Services Engineering, The Hong Kong Polytechnic University, Hung Hom, Kowloon, Hong Kong, China; beltw@polyu.edu.hk; 2 Department of Architecture, National Taiwan University of Science and Technology, Keelung Road Section 4, Taipei 106335, Taiwan; CCL@mail.ntust.edu.tw; 3 Department of Health Technology and Informatics, The Hong Kong Polytechnic University, Hung Hom, Kowloon, Hong Kong, China; polly.hm.leung@polyu.edu.hk

**Keywords:** drainage stack, experiment, positive air pressure, pathogen transmission, COVID-19, high-rise

## Abstract

Time-variant positive air pressure in a drainage stack poses a risk of pathogenic virus transmission into a habitable space, however, the excessive risk and its significance have not yet been sufficiently addressed for drainage system designs. This study proposes a novel measure for the probable pathogenic virus transmission risk of a high-rise drainage stack with the occurrence of positive air pressure. The proposed approach is based on time-variant positive air pressures measured in a 38 m high drainage stack of a full-scale experimental tower under steady flow conditions of flow rate 1–4 Ls^−1^ discharging at a height between 15 m to 33 m above the stack base. The maximum pressure and probabilistic positive air pressures in the discharging stack ventilation section with no water (Zone A of the discharging drainage stack) were determined. It was demonstrated that the positive air pressures were lower in frequency as compared with those in other stack zones and could propagate along the upper 1/3 portion of the ventilation pipe (*H’* ≥ 0.63) towards the ventilation opening at the rooftop. As the probabilistic positive pressures at a stack height were found to be related to the water discharging height and flow rate, a risk model of positive air pressure is proposed. Taking the 119th, 124th, 140th and 11,547th COVID-19 cases of an epidemiological investigation in Hong Kong as a baseline of concern, excessive risk of system overuse was evaluated. The results showed that for a 20–80% increase in the frequency of discharge flow rate, the number of floors identified at risk increased from 1 to 9 and 1 to 6 in the 34- and 25-storey residential buildings, respectively. The outcome can apply to facilities planning for self-quarantine arrangements in high-rise buildings where pathogenic virus transmission associated with drainage system overuse is a concern.

## 1. Introduction

Aerosols generated by discharging wastewater through indoor drainage stacks are among the suspected sources of virus transmission [1]. Based on the spread of COVID-19 virus aerosols from the drainage stack to the toilet of an apartment through a faulty connection to the stack, health risk associated with faulty connections to a building drainage stack was estimated [2]. To reduce the risk of pathogenic transmission such as SARS and SARS-CoV-2, understanding of positive air pressure in a vertical drainage stack has been identified as an important factor [3]. However, the excessive risk and its significance have not yet been taking account for drainage system designs. Studies showed that SARS-CoV can survive in the sewage for days to weeks [4]. The presence of SARS-CoV-2 in stools and wastewater was reported and a possible faecal-oral transmission was raised [5]. Defects in the wastewater plumbing system as a transmission mode within the building facilitated the transport of virus-laden droplets through empty u-bends or damaged drainage pipes in bathrooms [6]. Aerosols generated from contaminated drainage stack can generate transmission routes with positive air pressure in them. A contaminated faulty sewage system in a high-rise housing estate in Hong Kong in 2003 was linked to the SARS outbreak which involved a large number of residents living in the surrounding buildings [7]. Therefore, air pressure associated with a discharging stack should be investigated.

**Figure 1:**
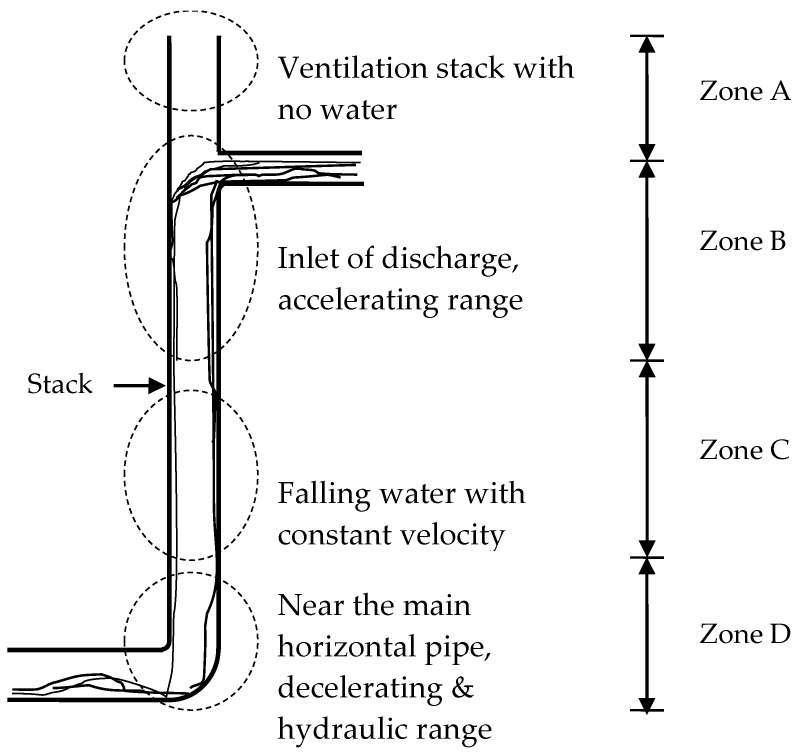
Four zones in a discharging drainage stack.

The spatial variation of average maximum air pressure can be described by a vertical zonal model [8]. Taking the discharge entrance at a level above the vertical stack base as a reference, there are 4 zones in this zonal model as shown in Figure 1. ‘Zone A’, the upstream part of the vertical stack vent from the discharge entrance, consists of airflow only and has an average pressure below atmospheric pressure due to the friction from an upstream pipe with an incoming airflow. ‘Zone B’ is located between the discharge entrance and the downstream point of atmospheric pressure where the air pressure recovers after reaching its negative peak. Discharges in this region accelerate until reaching a constant velocity. ‘Zone C’, in which airflow gains energy as a form of static pressure increment through deceleration against the falling water, is the downstream part of ‘Zone B’ with positive average air pressure. Falling water with constant velocity has been reported in this zone. ‘Zone D’ is located at the connection point to the horizontal pipe. The study developed empirical expressions approach to average peak air pressure in vertical drainage stacks given a single point discharge and steady flow conditions. 

**Figure 2:**
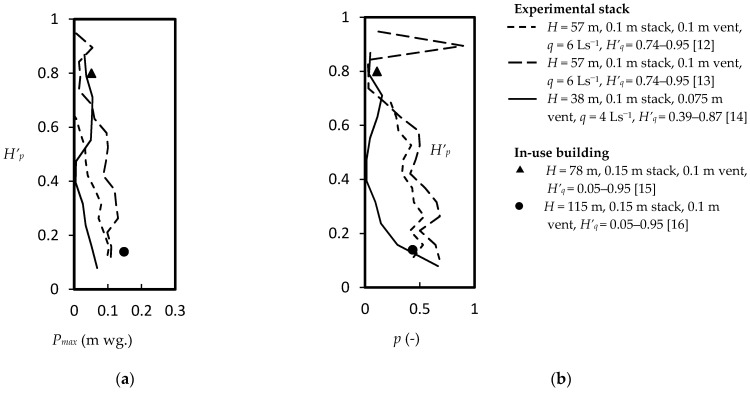
Positive air pressures within the discharging drainage stacks (*H =* 38–115 m) of an experimental tower and some in-use buildings: (**a**) maximum air pressure; (**b**) probability of positive air pressure.

In a discharging drainage stack, the flow regime comprises an annular water flow entraining a central air core with an associated pressure drop. Experimental evidence showed an unexpected distribution of water mass across the stack discharge which, together with the nature of the air-water interface, governs the stack air movement [9,10,11]. Time-averaged pressure and its positive peak values were measured in real scale high-rise experimental facilities [12,13,14] and some in-use buildings [15,16]. These measurements covered a wide range of stack heights (*H* = 57–115 m), drainage stack diameters (0.1–0.15 m), secondary ventilation stack sizes (0–0.1 m in diameter), and stack water discharging flow rates (*q* = 1–17.5 L/s) at various discharging heights (full stack height *H’_d_* = 0.05–0.95). Figure 2 plots the occasional positive air pressures recorded within the discharging drainage stacks [12,13,14,15,16]. In Figure 2b, the probability of positive pressure is the fractional counts of all positive pressure values measured in a 24-h period for unsteady flow conditions [15,16]. For measurements made under steady flow conditions, the probability was estimated using the reported minimum pressures at the 99% confidence intervals in measurement time periods of 140 s–300 s [12,13,14].

The focus of these existing studies was on time-averaged negative air pressure and likely water seal failure that occurred at some heights (Zones B & C) below the discharge locations [12,13,14,15,16]. Indeed, the most current design guides based on an over-simplified steady-state probabilistic approach that disregards the unsteady and transient nature of discharging wastewater flow [17,18,19]. The occurrence of probabilistic positive air pressure in Zone A and the associated excessive risk are not properly addressed in many drainage system designs.

The risk of pollutants spreading from a drainage stack to the environment is associated with positive air pressure in the stack. This study focuses on time-variant probabilistic positive air pressures in the discharging stack ventilation section with no water (Zone A of a discharging drainage stack) and proposes a novel measure for the probable pathogenic virus transmission risk associated with the drainage stack. This study aims to address existing research gaps of excessive risk estimates for a high-rise drainage stack with the occurrence of positive air pressure. This information is critical for understanding the spread of infectious diseases in designing water and drainage regulations.

## 2. Materials and Method

**Figure 3:**
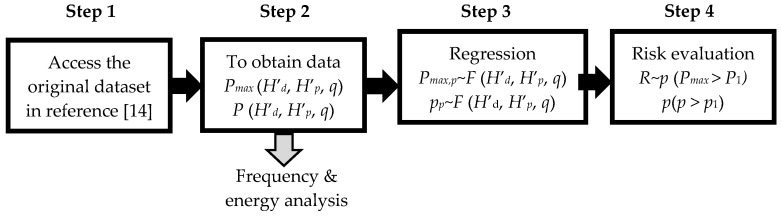
Development of risk index *R* for pathogen transmission risk in a contaminated drainage stack.

Figure 3 shows the materials and method adopted to obtain an indicative risk index for pathogen transmission risk in a contaminated drainage stack. The development includes 4 steps as shown in the figure and outline below.

(Step 1) Measurement data in a previous study [14] on the time-variant air pressure fluctuation characteristics in a 0.1-m of diameter high-rise drainage stack under steady flow conditions of flow rates from 1 Ls^−1^ to 4 Ls^−1^ discharging at a height between 15 m to 33 m above the stack base was used for this study. It was reported by Pink that the steady flow conditions covered the maximum ventilation flow rate require for discharging stacks (34–104 m of height, 0.1–0.15 m of diameter) [20]. These flow rates were typical for drainage stacks in high-rise buildings [21,22]. 

The experiment conditions in the previous study [14] were outlined below. Measurements were conducted in a full-scale experimental tower located at the National Taiwan University of Science and Technology (NTUST). The tower, with 13 levels above ground, was approximately 39 m tall. In brief, the testing facilities consisted of a main vertical drainage stack (100 mm in diameter and 38 m in height measured from the stack base) connected to a second ventilation stack (75 mm in diameter) using 50 mm slope vents. Water discharge points for levels 6–12 (i.e., at 15,18,21,33 m measured above the stack base) were served by a re-circulatory water supply. The pipework, with airflow measured in the upper region of the stack through a hot wire anemometer, was acrylic. Eleven pressure sensors were evenly distributed along with the main stack from levels 2–12 for air pressure recording (i.e., the height of pressure measurement *H* = 3,6,9…33 m above the stack base). Each pressure sensor (Unipulse, Tokyo, Japan) was factory calibrated which can measure air pressure up to ±5000 Pa with a resolution of 1 Pa and frequency response up to 1 kHz. The signal noise of the sensor was measured in still air and the high-frequency low-level noise signals were separated from the measured data by a low-pass signal threshold filter. 

(Step 2) In the measurements, with a sampling frequency of 0.01 s for a period of 30 s, the positive air pressure *P*~*P*(*H*’*_d_*, *H*’*_p_*, *q*) and its probability (occurrence) *p*~*p*(*H*’*_d_*, *H*’*_p_*, *q*) measured at a height *H_p_* (levels 2–12), along with the discharge points *H_d_* (at levels 6–12) at steady flow rates *q* = 1 Ls^−1^, 2 Ls^−1^, 3 Ls^−1^ and 4 Ls^−1^, are expressed in Equation (1) as a normalized stack height *H’* for generality [14].

(1) $$H_{p}^{\prime }=\frac{H_{p}}{H};\;H_{d}^{\prime }=\frac{H_{d}}{H}\;,$$

The potential energy regarding water seals connected to the stack at a height *E* (J kg^−1^) at time period *t_a_* (s) is given by Equation (2), where *P* (m wg.) is the gauge air pressure, *g* (=9.81 ms^−2^) is gravity, *t* (s) is the period of a pressure variation cycle, and *f* (Hz) is the frequency given by Equation (3), for *k* = 0, 1, …, *N* − 1 [23,24].

(2)  E=gP,

(3) $$f_{k}=\sum_{n=0}^{N-1}Pe^{-i2kn/N};\;k=0\ldots ,N-1;\;\;t=f^{-1},$$

(Step 3) Higher occurrence of maximum air pressure *P_max_* and the probability of positive air pressure *p* in a contaminated drainage stack can be an indicator of higher pathogen transmission risk through the stack. Both the maximum positive air pressure *P* and the probability *p* of having positive air pressure are assumed correlated to the heights *H*’*_p_*, *H*’*_d_* and the flow rates *q* [25] as shown in Equation (4).

(4) $$p,\;P_{max}\sim F\left(H_{p}^{\prime },\;H_{d}^{\prime },q\right),$$

Correlation between the outcome variable (*P_max_*, *p*) and the predictors (*H*’_d_, *H*’*_p_*, *q*) can be measured by the sample correlation coefficient *r* and test to be significant for *p* ≤ 0.05 of *t*-test *T* given by Equation (5) [26].

(5) $$T=\frac{r\sqrt{N-2}}{\sqrt{1-r^{2}}}$$

Linear regression is proposed to predict the average maximum positive air pressure *p* ≥ 0 and *P_max_* ≥ 0 in Equation (4) with the significant predictors, where *H’_p_* is the fractional stack height, *q* is the discharging flow rate and (*H’_d_*–*H’_p_*) is the fractional discharging height. The regression constants are determined by least-square fitting as shown in Equations (6) and (7) [26].

(6) $$P_{max,\;p}=0.01H_{p}^{\prime }+0.017\left(H_{d}^{\prime }-H_{p}^{\prime }\right)+0.009q-0.013$$

(7) $$p_{p}=0.138H_{p}^{\prime }-0.152\left(H_{d}^{\prime }-H_{p}^{\prime }\right)+0.069q-0.191$$

In Equations (6) and (7) the discharging flow rate *q* is taken as the probably maximum simultaneous demands in the stack given by Equation (8), where *q*_1_ is the flow rate of the appliance, *k* is the frequency factor of probabilistic discharge of appliances being connected to the stack [17],

(8) $$q=k\sqrt{\sum q_{1}},$$

(Step 4) In this study, a risk index *R* is proposed for a stack design at selected levels of the positive air pressure *P_max_* > *P*_1_ and probability *p* > *p*_1_ as shown in Equation (9), where *P*_1_ and *p*_1_ are respectively the reference positive air pressure and the reference probability of having positive air pressure of the transmission risk concerned.

(9) $$\;R\sim p\left(P_{max}>P_{1}\right)p\left(p>p_{1}\right);\;P_{max}=\max\left(P\right),$$

In Equation (9), the occurrence of positive air pressure *P* is assumed to be probabilistic and the probability *p* of having positive air pressure is determined by Equation (10) below, where *n*(*P* > 0) is the number of data acquired having an air pressure above ambient pressure and *n* is the total number of data in the same measurement period.

(10) $$\;p(P>0)=\frac{N\left(P>0\right)}{N},$$

## 3. Results and Discussion

**Figure 4:**
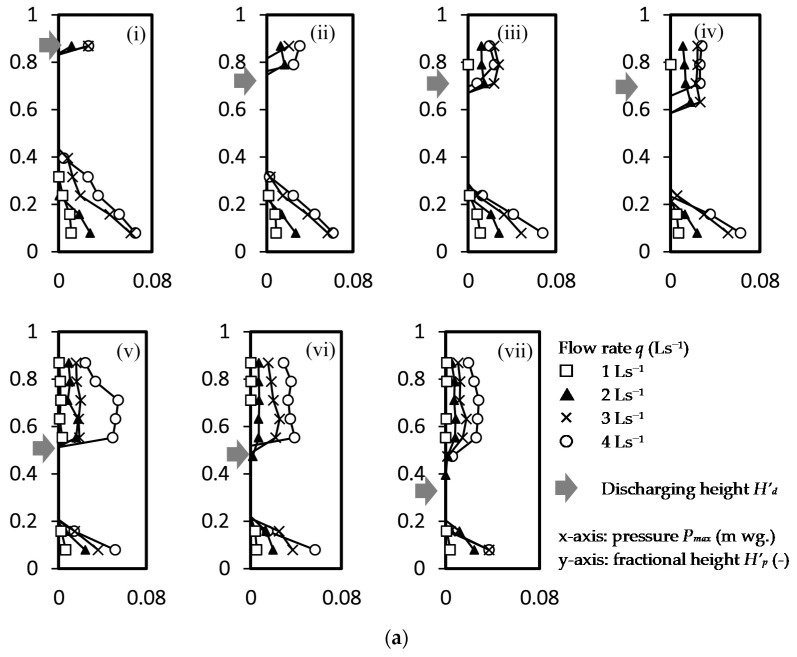

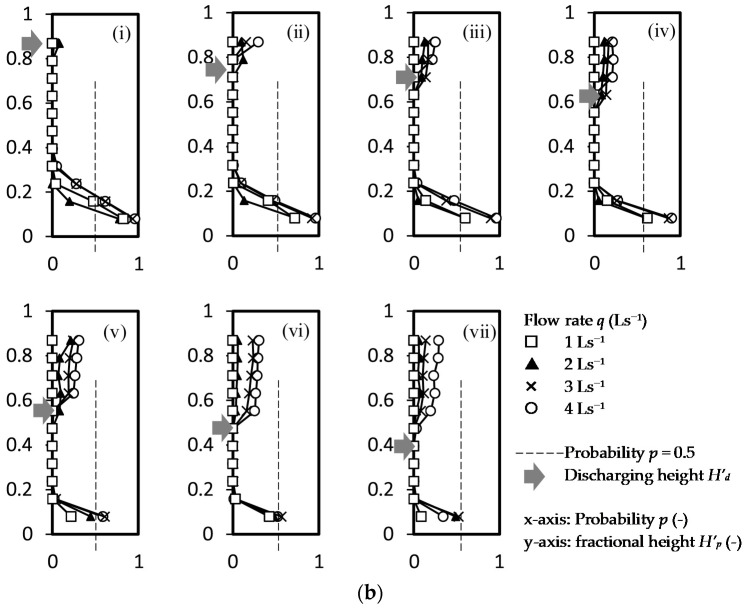
Positive air pressure within a discharging drainage stack (*H* = 38 m) at a normalized stack height *H’_p_* with a normalized stack discharging height *H’_d_*. (**a**) Maximum air pressure; (**b**) probability of positive air pressure.

The maximum positive air pressure *P_max_* (m, water gauge pressure of denoted as ‘m wg’) along a normalized stack height *H’_p_* (=0.08, 0.16, 0.24, 0.32, 0.39, 0.47, 0.55, 0.63, 0.71, 0.79, 0.87) for a steady flow rate *q* (=1, 2, 3, 4 Ls^−1^) discharged at 7 normalized height *H^’^_q_* (=0.39, 0.47, 0.55, 0.63, 0.71, 0.79 & 0.87) is shown in Figure 4a, where *H* (=38 m) is the stack height, *H_q_* (m) is the discharge height and *H_p_* (m) is the height of pressure measurement. Results of 28 cases (4 discharging flow rates at 7 discharging locations) were presented.

Apart from back air pressure measured near the stack base, positive air pressure was recorded occasionally at a height above the discharging height and this zone (Zone A) can be known as the local positive air pressure zone. These local positive air pressure values were found unsteady and their magnitude was in the range between 35%–75% (except one case got 106% in Figure 4a(v) of the back air pressure values. In these zones, two points were observed in a previous study [14]. First, peak pressure was on the negative side in all test cases, i.e., |−*P_min_*| > *P_max_*. Second, the time average air pressures over the measurement period were negative in all cases.

In Figure 4b, the occurrence of positive air pressure in this zone was probabilistic and probability *p* of having the positive air pressure is below 0.5 are shown in Figure 4b. In contrast, in the back pressure region (Zone D), the average air pressure was not lower than the ambient air pressure at a probability of 0.5 or above in most of the cases, except some cases of water discharging at a height *H’_p_* ≤ 0.55 at a water discharge flow rate of ≤2 Ls^−1^.

**Figure 5:**
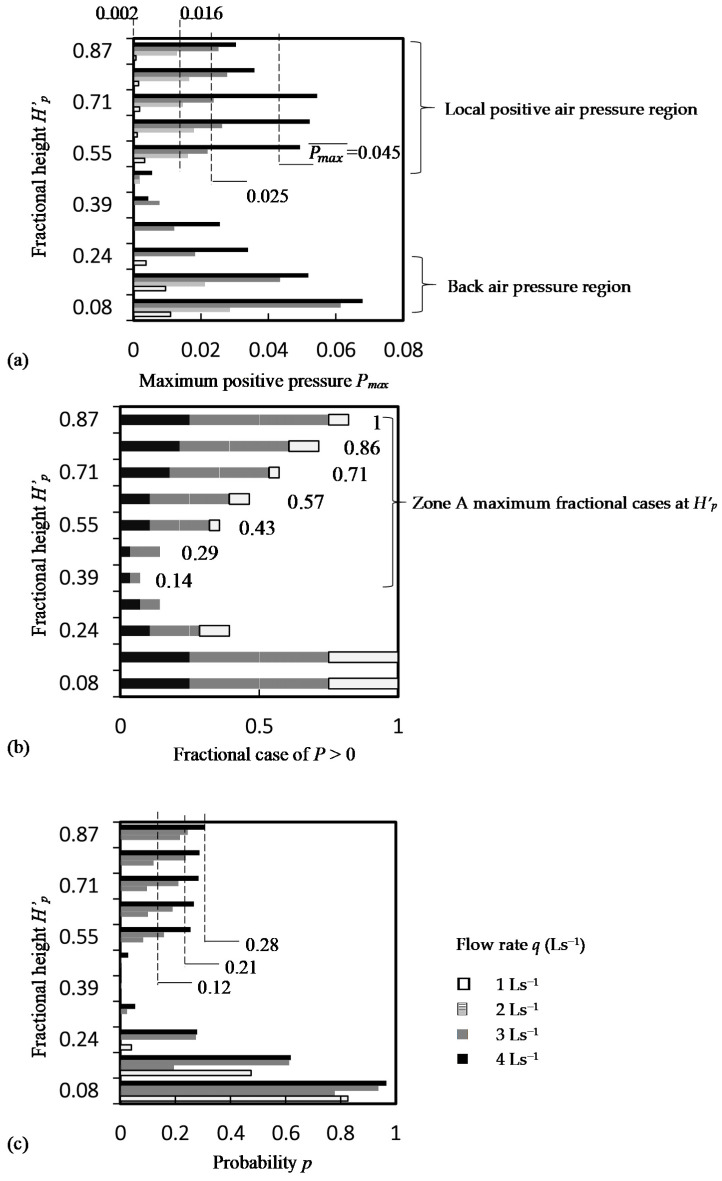
Positive air pressure occurrence profiles along with a drainage stack (*H* = 38 m). (**a**) Maximum positive pressure (m wg.); (**b**) fractional cases having positive pressure; (**c**) fractional discharging time having positive pressure in a case.

Figure 5a shows the maximum air pressure recorded at a stack fractional height *H’_p_*. The fractional number of 28 test cases having positive air pressure recorded is shown in Figure 5b. The average maximum local positive air pressure did not vary significantly in Zone A, except for the test condition discharging the highest flow rate of 4 Ls^−1^. The average maximum pressure is from 0.002(±0.000) to 0.045(±0.005) m wg for a discharge flow rate of 1 to 4 Ls^−1^ at 0.55 ≤ *H’_p_* ≤ 0.87.

Apart from the back air pressure region that all cases would generate positive pressures, cases of positive air pressures were found increasing with the fractional height *H’_p_* in Zone A. Probability of positive air pressure at Zone A was calculated by the fractional case in Figure 4b divided by the maximum fractional cases (numeric values are shown in the figure) were 0.82 (±0.01) at 0.55 ≤ *H’_p_* ≤ 0.87 and 0.5 (±0.00) at 0.39 ≤ *H’_p_* ≤ 0.47.

Figure 5c shows the probability of having positive air pressure determined by the measurement period. It was observed in Zone A that *p* > 0.05 occurs only at *H’_p_* ≥ 0.55 and at a discharging flow rate of ≥ 2 Ls^−1^. Although positive air pressure was recorded at 0.39 ≤ *H’_p_* ≤ 0.47, the occurrence was not noticeable at a test flow rate < 4 Ls^−1^. The probability did not vary significantly in Zone A and was found linear increasing with the discharging water flow rate (*p* < 0.01, *t*-test) 1 Ls^−1^ ≤ *q* ≤ 4 Ls^−1^.

**Figure 6:**
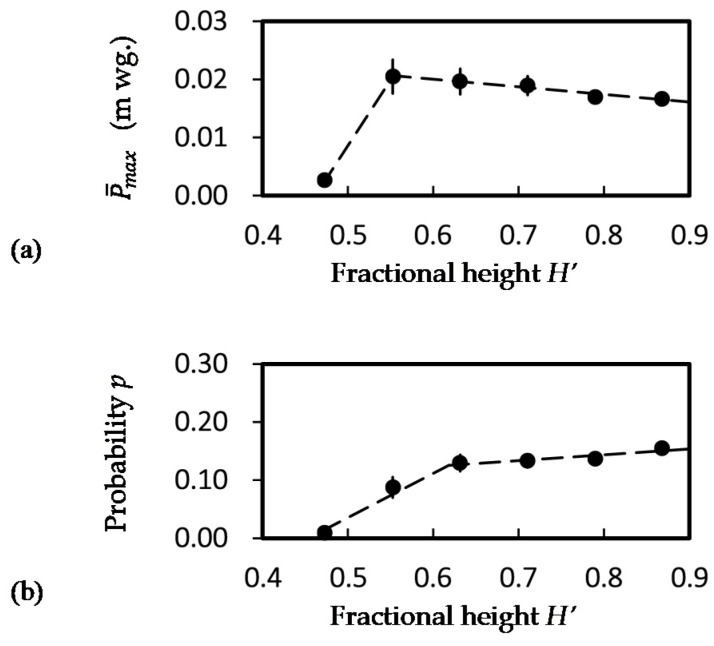
Measured quantity at a fractional height *H’* of a discharging stack. (**a**) Average maximum air pressure ${\bar{P}}_{max}$; (**b**) probability *p*.

Figure 6 shows at fractional height *H’* from all experience cases (a) the average maximum air pressure ${\bar{P}}_{max}$ (m wg.) and (b) the average probability *p*, with error bars indicated two standard errors. The average maximum air pressure at *H’_p_* ≥ 0.55 is 0.019 m wg., with a slight downtrend of 0.001 m wg. for an increasing height of 0.1 *H’* (*p* < 0.01, *t*-test). The average probability of positive pressure at 0.63 ≤ *H’_p_* ≤ 0.87 is 0.14 with a slight uptrend of 0.01 as for an increased height of 0.1*H’* (*p* < 0.1, *t*-test). The results indicated that the upper portion (*H’* ≥ 0.63) of a discharging stack would influence the positive air pressure propagation towards the ventilation pipe open at the roof (*H’* = 1).

**Figure 7:**
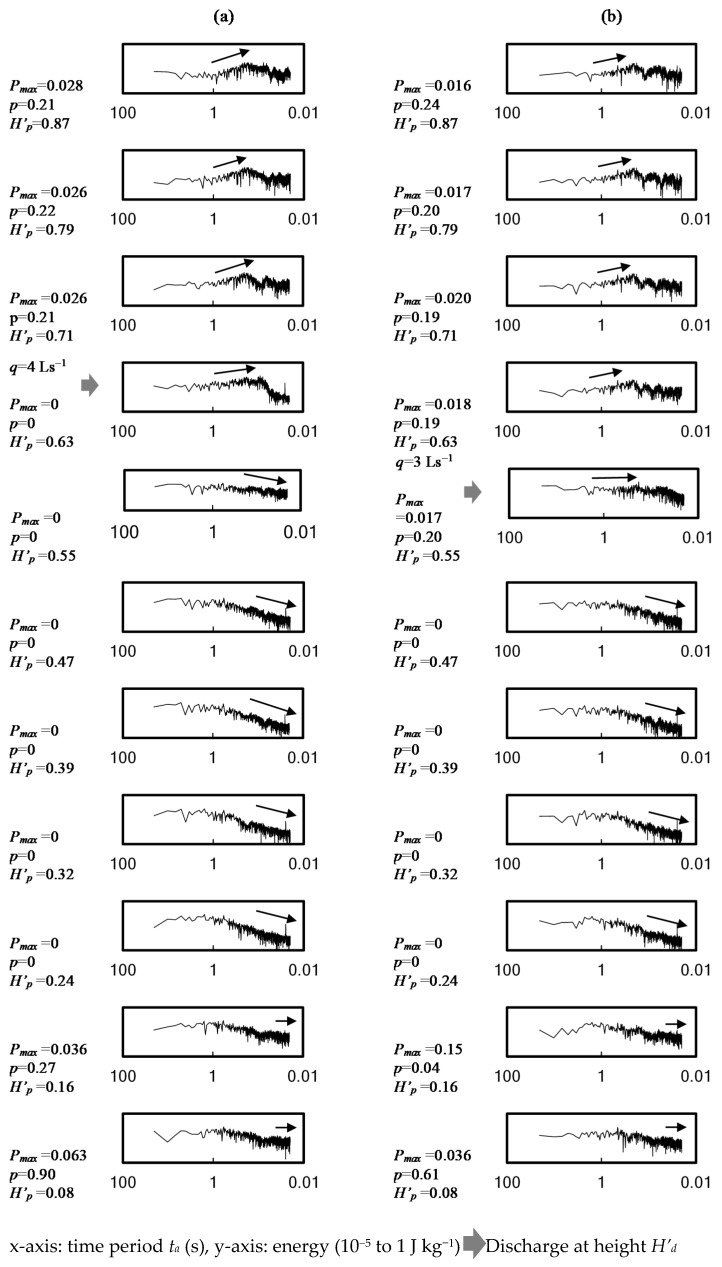
Energy spectrum density examples of air pressure along with a stack (measurement time = 20.48 s). (**a**) Discharge at *H’* = 0.63 at a flow rate of 4 Ls^−1^; (**b**) discharge at *H’* = 0.55 at a flow rate of 3 Ls^−1^.

Figure 7 exhibits example plots of energy spectrum density of time-variant air pressure fluctuations along with a stack, at a discharging flow rate *q* = 4 Ls^−1^ at *H’_d_* = 0.63 and *q* = 3 Ls^−1^ at *H’_d_* = 0.55 respectively. As the measurement period was 5 min at a sampling frequency of 0.01 s, the period *t* in the figure determined by the fast Fourier transformation was 0.02 s to 20 s. The figures showed that energy concentrate at *t* = 0.06−1 s in Zone A, as a result of discharging water flows, compressed air and air turbulent energy dissipated the stack. In Zones B and C, the figures showed a clear decreasing trend of energy from time 1 to 0.02 s. However, in Zone D, as a contrast to Zones B and C, the energy kept at a level over the range of *t* = 0.02−0.06 s. It was noted that positive air pressure was recorded in both Zones A and D. In Zone D, however, turbulent eddies were small, quickly dissipated and the positive air pressure influence was confined near the stack bottom part (*H’_d_* = 0.08−0.16). In Zone A, larger turbulent eddies would take a longer period of turbulent energy absorption by breaking down into smaller and smaller eddies. Thus the positive air pressure influence sustained for a longer, upper portion of a stack (*H’_d_* = 0.63−0.87 at *H’_q_* = 0.63 and *H’_d_* = 0.55−0.87 at *H’_q_* = 0.55), as shown in Figure 7a,b.

The average maximum positive air pressure and its occurrence probability of a discharging stack at *H’* ≥ 0.47 were found to be related to fractional stack height *H’_p_*, discharging flow rate *q* and fractional discharging height *H’_d_*–*H’_p_* (*p* < 0.05, *t*-test) by regression analysis [26] and Equations (6) and (7) were tested to be significant (*p* < 0.01, *t*-test).

**Figure 8:**
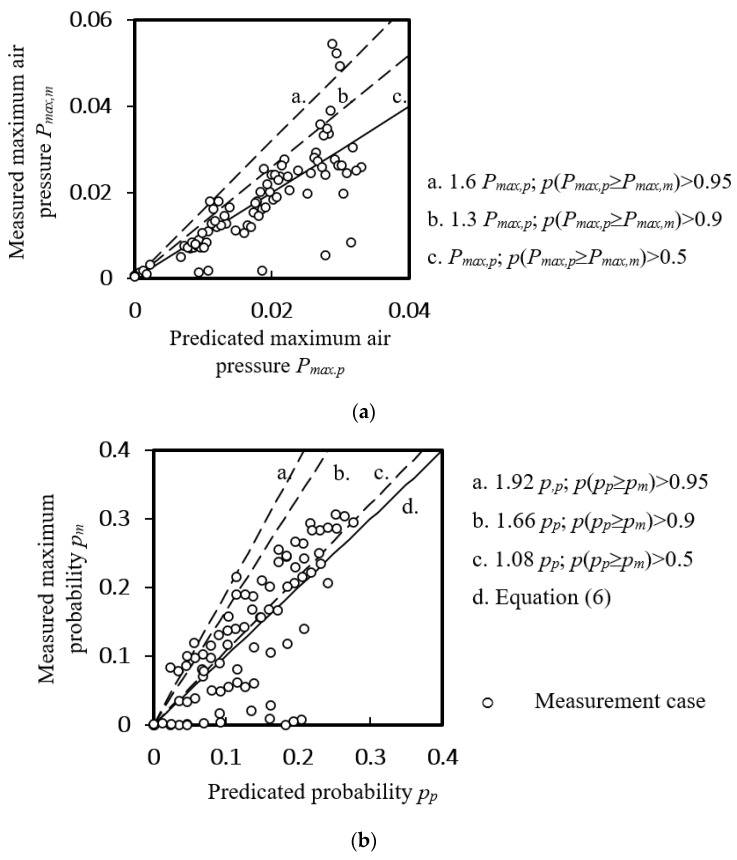
Predicted quantity at Zone A of a discharging drainage stack at *H’* ≥ 0.47. (**a**) Maximum air pressure; (**b**) the probability of positive pressure.

Figure 8 shows the predicated maximum air pressure and the probability of positive pressure against the measured ones in Zone A of a discharging drainage stack. About 50% of the measured maximum air pressures in Zone A were not exceeding the predicted values given by Equation (6). To give a safety margin of 90% and 95% coverage against the local positive air pressure, a constant of 1.3 and 1.6 for the pressure, 1.08, 1.66 and 1.92 for the probability is illustrated in the figures.

## 4. Application Examples

According to the Government of the Hong Kong Special Administrative Region Press Release dated 15 March 2020, the 140th COVID-19 case (case I) located on the 34th floor of a residential building was confirmed 3 days after another 2 confirmed cases (119th & 124th) found on the 32nd floor of the same building. The two units involved were sharing the same drainage stack. Among subsequent tests of 12 environmental samples collected at those two units and the rooftop, 4 of them were tested positive. Apart from the three samples collected near the water closets inside the two units, a sample taken from the open end of the rooftop vent pipe was also tested positive.

Another case (case II), where two cases (22/F & 21/F) were affirmed after a confirmed case (11,547th) located on the 23rd floor of a residential building, was reported on 9 April 2021. All the units involved were sharing the same drainage stack.

The experimental results could supplement the epidemiological investigation findings. First, both units’ WC in case I were connected to the same stack (*H’_p_* = 0.85–0.9) where unsteady local positive air pressure was recorded in a discharging stack experimentally at *H’_p_* ≥ 0.55. Second, 34/F unit’s WC was connected to the stack Zone A when 32/F unit’s WC discharging. Later confirmed case and tested positive samples were reported above 32/F (Zone A) but not floors below it (Zone B). As the experiments recorded local positive air pressure only in Zone A but not in Zone B.

A similar situation was observed in case II where all of the WCs involved were connected to the same stack (*H’_p_* = 0.80–0.88). Discharges at a lower level (i.e., 20/F and below) into a stack contaminated by pathogens generated local positive air pressure (Zone A) and posed excessive transmission risk to the upper levels (i.e., 21/F & 22/F).

**Figure 9:**
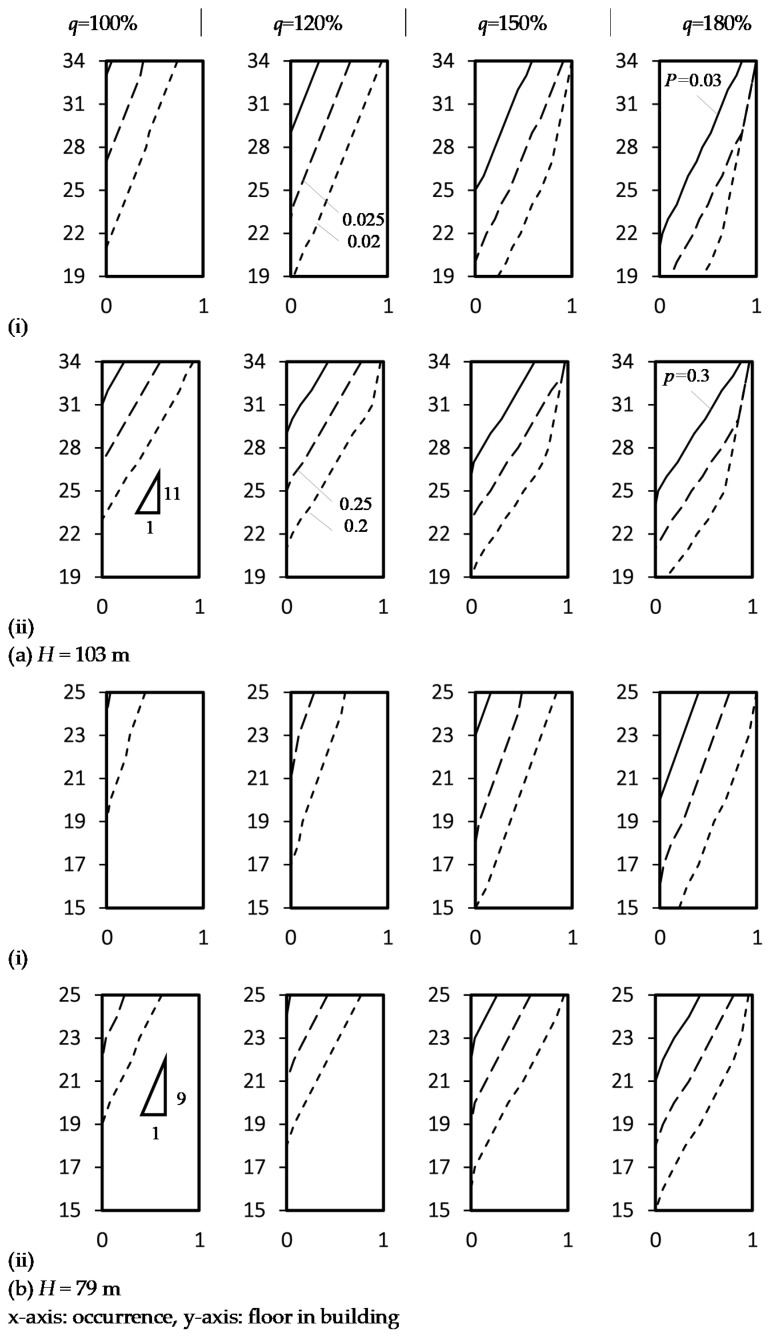
Zone A positive air pressures and occurrences inside the stacks of two high-rise residential buildings with confirmed samples of COVID-19 virus. (**a**) Height *H* = 103 m; (**b**) height *H* = 79 m; (**i**) maximum positive air pressure; (**ii**) probability of positive pressure.

Figure 9 illustrates the Zone A positive air pressures and occurrences inside the stacks of two Hong Kong high-rise residential buildings with confirmed samples of the COVID-19 virus. Supposed all floors were occupied and WCs could be discharged occasionally. Follow some design practices of WC, taking an example flow rate of one WC of *q*_1_ = 1.8 L/s, the probably maximum simultaneous demands in the stack calculated by Equation (8) were used as the discharging flow rate in Equations (6) and (7) for the floor levels of *H’_p_* & *H’_d_*, with *k* = 0.5 the frequency factor for intermittent use (typical dwelling, guesthouse and office) [27,28]. The design flow rate *q* for intermittent use allows each installed WC to discharge once in every 20 min in the daily rush hour [27,28]. For frequent use like hotel, restaurant, school and hospital, *k* = 0.7, taking account of the discharge once every 10 min, is adopted.

Figure 9(ai,bi) shows the occurrence of predicted probabilistic positive air pressures at 0.02 ≤ *P_max_* ≤ 0.03 and 0.2 ≤ *p* ≤ 0.3. Higher pathogen transmission risk was identified for higher floors where the higher occurrence of positive air pressure was predicted as shown in Figure 9ii. Taking a reference risk level of pressure *P* (=0.025 m wg.) for a stack height *H* = 103 m at the nominal *q* (=100%), the occurrences of *p* (=0.25) on 33/F and 32/F are 0.51 and 0.42 respectively (Figure 9aii). Approximately, the unit occurrence of *p* covers 23/F to 34/F (i.e., 11 floors; 0.09 per floor) for *H =* 103 m or 16/F to 25/F (i.e., 9 floors; 0.11 per floor) for *H* = 79 m. Higher transmission risk was identified for a taller stack and thus the top floors. Occurrences of *p* (=0.25) at *P* (=0.025 m wg.) predicted for the topmost floor were 0.6 and 0.23 for *H =* 103 m and 79 m respectively.

**Figure 10:**
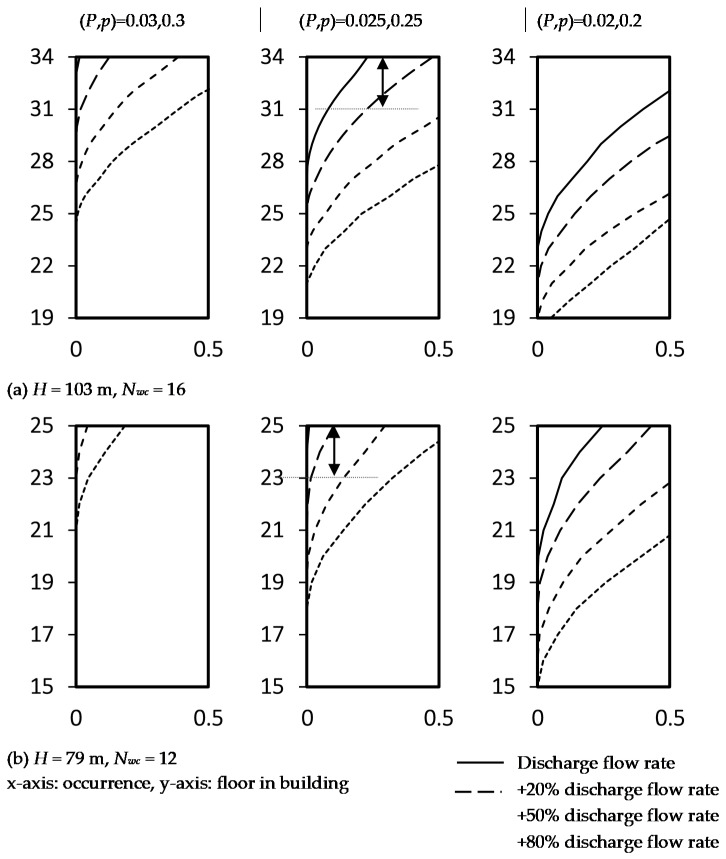
Sensitivity risk increment due to 20% more discharge flow rate in two example buildings. (**a**) Height *H* = 103 m; (**b**) height *H* = 79 m.

Self-isolation can lead to a greater number of infected people in a building and potential system overuse. It is a matter of concerns of self-quarantine arrangement at home for 2–3 weeks and more frequent discharge of WC lead to a higher discharging flow rate in a stack. For WCs uses of extra discharge frequency up to 100%, each WC would discharge in a manner of once in every 10–20 min in the daily rush hour. The corresponding frequency factor *k* = 0.7 [17,27,28]. Figure 10 shows the sensitivity of the risk indicators for a case of a 20%–80% (*k* = 0.55–0.67) extra discharge frequency of WCs in two example buildings (*H =* 79 m and 103 m).

Take the case on the topmost floor as a reference, the reference risk values at selected levels of positive air pressure and probability (*P =* 0.025 m wg., *p* = 0.25) are 0.23 (*H* = 103 m) and 0.09 (*H* = 79 m) as shown in Figure 10. Corresponding to the additional 20%, 50% and 80% in discharge flow rate expected at *H =* 103 m, three, six and nine more floors (i.e., 31/F to 33/F, 28/F to 33/F and 25/F to 33/F) will be identified at risk if the reference risk value is taken as a baseline of concern as shown in Figure 10a. For *H* = 79 m, two, four and six more floors will be at risk as shown in Figure 10b. In the planning of self-quarantine facilities in high-rise buildings where the transmission of pathogenic viruses associated with overuse of the drainage system is a concern, floors at an excessive risk increment should not be occupied with the examples demonstrated in Figure 10.

## 5. Conclusions

This study proposed a novel risk index of the probable pathogenic virus transmission risk for a high-rise drainage stack with the occurrence of positive air pressure and examined the characteristics of time-variant positive air pressures in Zone A of a discharging stack, i.e., the ventilation stack section with no water. Positive pressure generated at the height of discharged water in the stack could propagate along the upper 1/3 portion of the ventilation pipe (*H’_p_* ≥ 0.63) towards the ventilation opening at the rooftop. The average maximum positive pressure and the probability of positive pressures were found to be related to the water discharging height and flow rate, and mathematical expressions were proposed to indicate excessive risk due to the positive air pressures. Based on the epidemiological investigation reported for the 119th, 124th, 140th and 11,547th COVID-19 cases in Hong Kong, the baseline risk was taken as the positive air pressure inside the involved units. An excessive risk of drainage system overuse was evaluated. It was demonstrated that with an additional 20–80% in the frequency of discharge flow rate, the number of floors identified at risk increased from 1 to 9 and 1 to 6 in the 34- and 25-storey residential buildings respectively. The results would be useful for understanding the effects of positive air pressure on trap seals connected to a drainage stack in a high-rise building and for measuring the probabilistic positive air pressure in high-rise drainage systems. It would also be useful for facilities planning of self-quarantine arrangements in high-rise buildings where pathogenic virus transmission associated with drainage system overuse is a concern. Nevertheless, these estimates were made based on the results measured in a 100-mm diameter, 38-m tall drainage stack with a ventilation stack of 50 mm in diameter. Despite full-scale experiments are resources demanding, further measurements in an extended range of stack heights are required to reduce the uncertainty of predictions.

## Data Availability

The data presented in this study are available on request from the first author.

## Nomenclature

*E*	unit potential energy (J kg^−1^)
*F*()	function of
*f*	frequency (Hz)
*f_k_*	Fourier transformation at frequencies *n* = 0, 1, 2,…, *n* − 1 and *k* = 0, 1, 2,…, *n* − 1
*g*	gravity (=9.81 ms^−2^)
*H, H’*	stack full height (m), normalized stack full height *H’* = 1
*H_d_, H’_d_*	stack discharging height (m), normalized stack discharging height (-)
*H_p_, H’_p_*	stack height of air pressure measurement (m), normalized stack height of air pressure measurement (m wg.)
*k*	frequency factor
max	maximum value
*n*	number of samples
*p*	probability (-)
*P*	air pressure (m wg.)
$\bar{P}$	average air pressure (m wg.)
*q*	stack water discharging flow rate (Ls^−1^)
*R*	risk index defined in Equation (9) (-)
*r*	correlation coefficient (-)
*T*	T-statistics for *t*-test (-)
*t*	time period of a fluctuation cycle (s)
Subscript	
1	of reference condition of transmission risk concerned
*m*	of measured value
*max*	of maximum value
*p*	of predicted value
